# SIRT6-PAI-1 axis is a promising therapeutic target in aging-related bone metabolic disruption

**DOI:** 10.1038/s41598-023-33297-7

**Published:** 2023-05-17

**Authors:** Alkebaier Aobulikasimu, Tao Liu, Jinying Piao, Shingo Sato, Hiroki Ochi, Atsushi Okawa, Kunikazu Tsuji, Yoshinori Asou

**Affiliations:** 1 Department of Orthopedics Surgery, Tokyo Medical and Dental University, 1-5-45 Yushima Bunkyo-Ku, Tokyo, 113-8519 Japan; 2 Department of Rehabilitation for Movement Functions, Research Institute, National Rehabilitation Center for Persons With Disabilities, Tokorozawa-Shi, Saitama, Japan

**Keywords:** Senescence, Mechanisms of disease, Metabolic bone disease, Bone

## Abstract

The mechanistic regulation of bone mass in aged animals is poorly understood. In this study, we examined the role of SIRT6, a longevity-associated factor, in osteocytes, using mice lacking *Sirt6* in *Dmp-1*-expressing cells (cKO mice) and the MLO-Y4 osteocyte-like cell line. cKO mice exhibited increased osteocytic expression of *Sost, Fgf23* and senescence inducing gene *Pai-1* and the senescence markers *p16* and *Il-6*, decreased serum phosphate levels, and low-turnover osteopenia. The cKO phenotype was reversed in mice that were a cross of PAI-1-null mice with cKO mice. Furthermore, senescence induction in MLO-Y4 cells increased the *Fgf23* and *Sost* mRNA expression. *Sirt6* knockout and senescence induction increased HIF-1α binding to the *Fgf23* enhancer sequence. Bone mass and serum phosphate levels were higher in PAI-1-null aged mice than in wild-type mice. Therefore, SIRT6 agonists or PAI-1 inhibitors may be promising therapeutic options for aging-related bone metabolism disruptions.

## Introduction

Osteoporosis is a skeletal bone disease characterized by reduced bone mass, which results in increased fracture risk. Aging is a pivotal risk factor for osteoporosis. The yeast sirtuin SIR2 is an NAD^+^-dependent histone deacetylase that is involved in the regulation of aging^1^. In mammals, the sirtuin family contains seven proteins (SIRT1-SIRT7), each with different tissue specificity, intracellular localization, and enzyme activity. SIRT6 is expressed in the nucleus and plays a role in transcriptional silencing and maintaining genomic stability^2^. *SIRT6* deficient mice die young and display phenotypes of premature aging, including osteoporosis^2^. We previously showed that SIRT6 regulates chondrocyte proliferation and differentiation by regulating *Ihh* expression^3^. Furthermore, SIRT6 deficiency aggravates joint cartilage degeneration in a metabolic syndrome-associated osteoarthritis model^4^. SIRT6 is ubiquitously expressed cells, including osteoblasts and osteocytes, and SIRT6-null mice are characterized by low-turnover osteoporosis^5^. SIRT6 regulates bone metabolism in osteoblasts by binding to the promoters of *Runx2* and osterix and deacetylating histone H3 at lysine 9 (H3K9)^5^. However, SIRT6*-*null mice die young, so the precise role of SIRT6 in bone metabolism, especially in osteocytes, is poorly understood^6^.

Osteocytes are derived from osteoblasts and they play a central role in bone remodeling. Osteocytes can regulate bone turnover with the production of nitric acid, prostaglandins, RANKL, and sclerostin^7,8^. Furthermore, osteocytes play a pivotal role in the regulation of phosphate homeostasis by secreting FGF23. FGF23 is a potent phosphaturic peptide hormone that is predominantly secreted by osteocytes and osteoblasts^9^. FGF23 suppresses serum phosphate levels by inhibiting the reabsorption of proximal tubular phosphate and the absorption of intestinal phosphate. Serum levels of FGF23 increase with age in men aged over 60^10^. *FGF23* mRNA levels are significantly higher in older women (mean age of 72.9 years) than in young women (mean age of 30.0 years)^11^. Patients with chronic kidney dysfunction and aging-related renal disease exhibit higher serum FGF23 levels^12^.

Sclerostin, encoded by *SOST*, is secreted by differentiated osteocytes and inhibits canonical Wnt signaling and bone formation by interacting with LRP5^13–17^. *Sost*-null mice exhibit elevated bone volume as a result of enhanced Wnt signaling, whereas transgenic mice overexpressing *Sost* display reduced bone volume. The pivotal role of SOST in aging-related osteoporosis has been elucidated in rodent models^18^. When senescent cells are genetically depleted in mice, aging-related osteoporosis is prevented, and *Sost* expression is reduced^18^. Additionally, calorie restriction, which extends lifespan by activating the sirtuin family, suppresses *Sost* mRNA expression^19^.

Thus, although the involvement of FGF23 and SOST in aging-related skeletal disorders has been reported, the mechanisms by which the aging process affects FGF23 and SOST expression are poorly understood. Thus, we examined the role of SIRT6 in osteocytes by analyzing *Dmp-1*-expressing cells in *Sirt6*-deficient mice (*Dmp-1*cre::*Sirt6f*^*/f*^ mice; cKO). Furthermore, as *Sirt6* deficiency enhances *Pai-1* expression, PAI-1*-*null mice were crossed with cKO mice, generating cPKO mice (*Dmp-1*cre::*Sirt6f*^*/f*^::*Pai-1*−/− mice); the function of the *Sirt6*-*Pai-1* axis in osteocyte-related skeletal metabolism was examined.

## Results

### Evaluation of bone parameters in cKO mice

**Figure 1:**
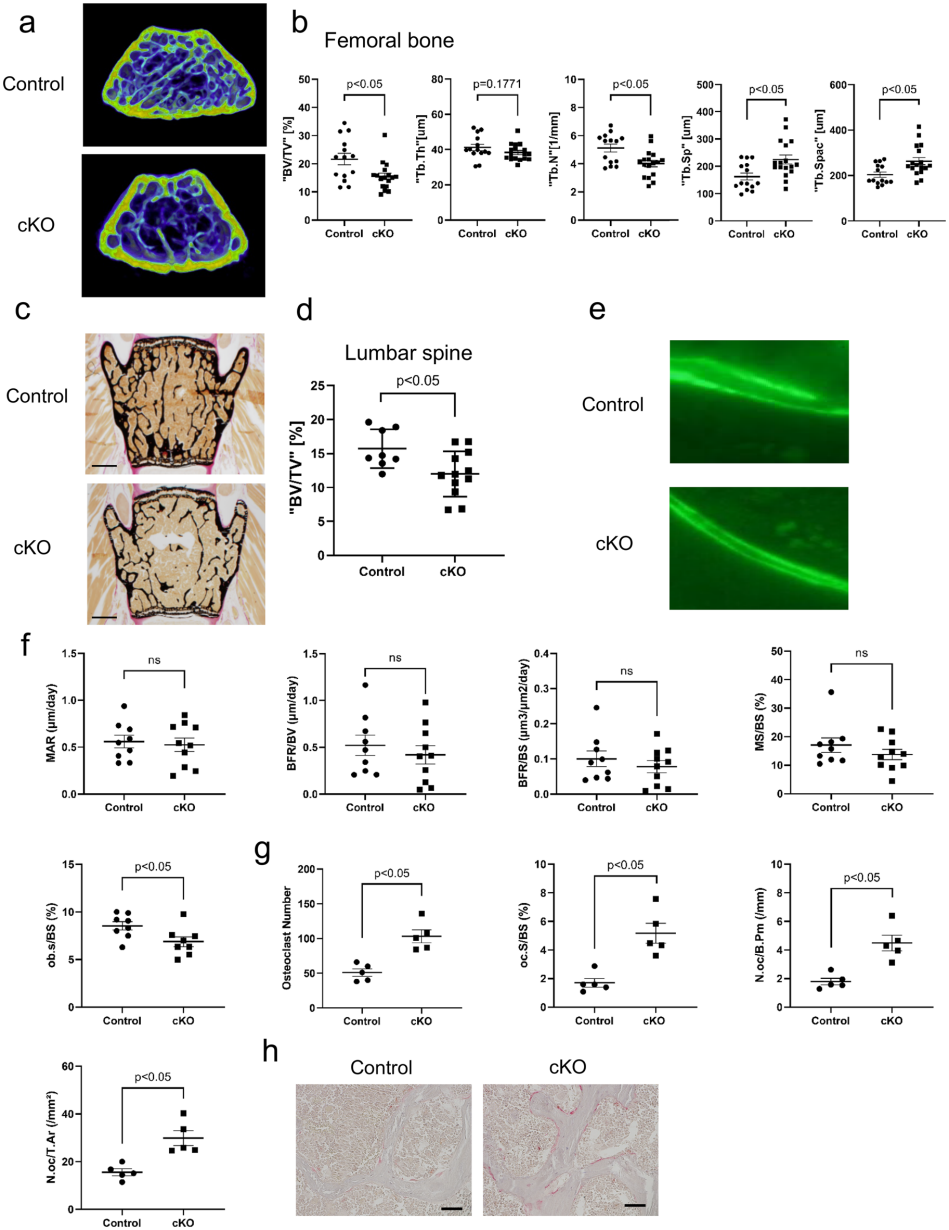
SIRT6 deficiency in osteocytes reduces trabecular bone volume (BV) of lumbar spine and distal femur. (a) Representative 3D micro-CT images of the distal femur region from male and female control and cKO mice. (**b**) Micro-CT analysis results for the femoral bone of control and cKO mice (*n* > 8 per group). (**c**–**g**) Histomorphometric analysis of lumbar vertebra trabecular bone. (**c**,**d**) Trabecular bone phenotype in lumbar vertebra. (**c**) Representative images of lumbar spine vertebra (L3) from male control and cKO mice (scale bar, 1 mm). (**d**) Average BV/TV values taken from L3 and L4 vertebra. (**e**) Fluorescence micrograph of the trabecular bone section showing calcein labeling (green). (**f**) Quantification of *MAR*, *BFR/BS*, *MS/BV*, *MS/BS* and *ob/BS.* (**g**) Quantification of tartrate-resistant acid phosphatase (TRAP)‐stained osteoclast surface and osteoclast number. (**h**) Representative images of TRAP‐stained sections from control and cKO mice (scale bar, 50 μm). Data represent mean ± SD for eight mice/group. *P < 0.05. *cKO* DMP1cre::*Sirt6f.*/f mice; *BV/TV* bone volume/tissue volume; *Tb.th* trabecular bone thickness; *Tb.sp* trabecular bone separation; *Tb.Spac* Trabecular bone spacing; *Tb.n* trabecular bone number; *MAR* mineral apposition rate; *BFR/BV* bone formation rate/bone volume; *BFR/BS* bone formation rate/bone surface; *MS/BS* mineralizing surface/bone surface; *ob.S/BS* osteoblast surface/bone surface: *oc.S/BS* osteoclast surface/bone surface; *N.oc/B.Pm* osteoclast number/bone perimeter; *N.oc/T.Ar* osteoclast number/tissue area.

The cKO mice grew normally, and the body weights of the cKO mice and littermates were similar (Supplementary Fig. 1). Bone volume was evaluated in the lumbar and femoral bones. At 20 weeks after birth, male cKO mice exhibited lower trabecular bone volume in the femur and lumbar spine than the control mice (Fig. 1a–d). Micro-computed tomography (micro-CT) analysis of the femoral bone indicated higher trabecular separation (Tb.Sp) and trabecular bone spacing (Tb.Spac) in cKO mice compared to the control mice, whereas the trabecular number (Tb.N) was lower in cKO mice than in the control mice (Fig. 1b). Bone histomorphometry of the lumbar spine indicated that osteoblast surface to bone surface (ob. s/BS) ratio was significantly decreased in cKO mice. The mineralizing surface to bone surface (MS/BS) ratio, bone formation rate (BFR)/BS, and mineral apposition rate (MAR) tended to be lower in cKO mice than in the control mice; however, the differences were not significant (Fig. 1e,f). The number of osteoclasts (osteoclast number to bone perimeter, osteoclast number to total area) and osteoclast surface (osteoclast surface to BS) increased in cKO mice (Fig. 1g,h). These data indicate that bone resorption activity was higher in cKO mice than in the control mice.

### Evaluation of bone parameters and serum phosphate levels in cPKO mice

**Figure 2:**
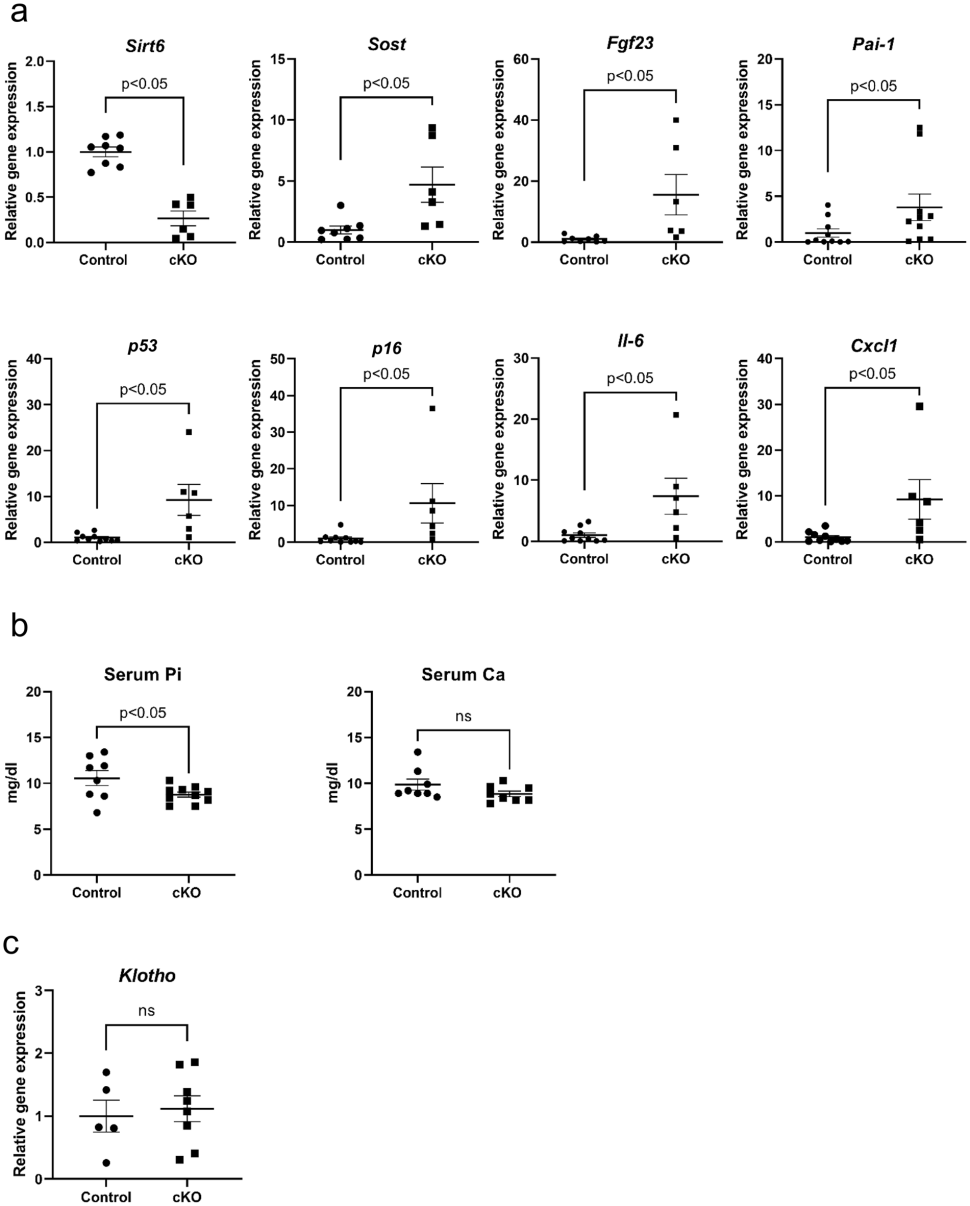
SIRT6 deficiency induces enhancement of *Sost* and *Fgf23* expression. (**a**) qPCR analysis of the indicated genes in the cortical bone of control and cKO mice. (**b**) Serum levels of phosphate and calcium in the control and cKO mice. (**c**) qPCR analysis of *klotho* gene in the kidney tissue of control and cKO mice. Data represent mean ± SD. *P < 0.05. cKO: DMP1cre::*Sirt6f.*/f mice.

To elucidate the molecular targets of SIRT6 in osteocytes, we assessed the mRNA expression of potent regulators of bone formation (*Sost*), bone resorption (*Opg* and *Rankl*), and *Fgf23* in osteocyte-enriched cortical bones. Quantitative polymerase chain reaction (qPCR) analysis indicated that the mRNA expression of *Sirt6* was knocked out in the cortical bone samples of cKO mice (Fig. 2a). mRNA of *Sost* and *Fgf23* was significantly increased in the tibial cortical bone of cKO mice (Fig. 2a), whereas differences between *Opg* and *Rankl* mRNA expression were not significant between cKO and the control mice (Supplementary Fig. 2). We previously revealed that SIRT6 deficiency enhanced the expression of *Pai-1*, a potent senescence marker, in chondrocytes^3^, so the mRNA level of *Pai-1* was also assessed. As expected, *Pai-1* mRNA expression also increased in cKO osteocytes (Fig. 2a). We then evaluated the expression of senescence marker genes, such as *p53*, and *p16* and the markers of senescence-associated secretory phenotype (SASP), interleukin-6 (*Il-6*), C-X-C motif chemokine ligand 1 (*Cxcl1*). qPCR analysis indicated that the expression of these genes was increased in cKO mice (Fig. 2a). In humans, FGF23 is a potent regulator of phosphate metabolism^9^. Therefore, serum phosphate levels were evaluated in cKO mice. As expected, Serum phosphorus levels were significantly lower in cKO mice compared to control mice, whereas, serum calcium levels were comparable in control and cKO mice (Fig. 2b). DMP-1 is also expressed in the distal tubules of the kidney^20^. The distal tubules express klotho, an important factor in phosphorus metabolism. To verify the involvement of the variation in klotho expression in the abnormal phosphorus metabolism observed in cKO mice, we compared the expression of klotho between controls and cKO by q-PCR (Fig. 2c). The results showed no significant difference in klotho expression between control and cKO, suggesting that phosphorus metabolism in cKO mice was due to increased FGF23 expression from osteocytes.

### Immunohistochemistry analysis of cortical bone in cKO mice

**Figure 3:**
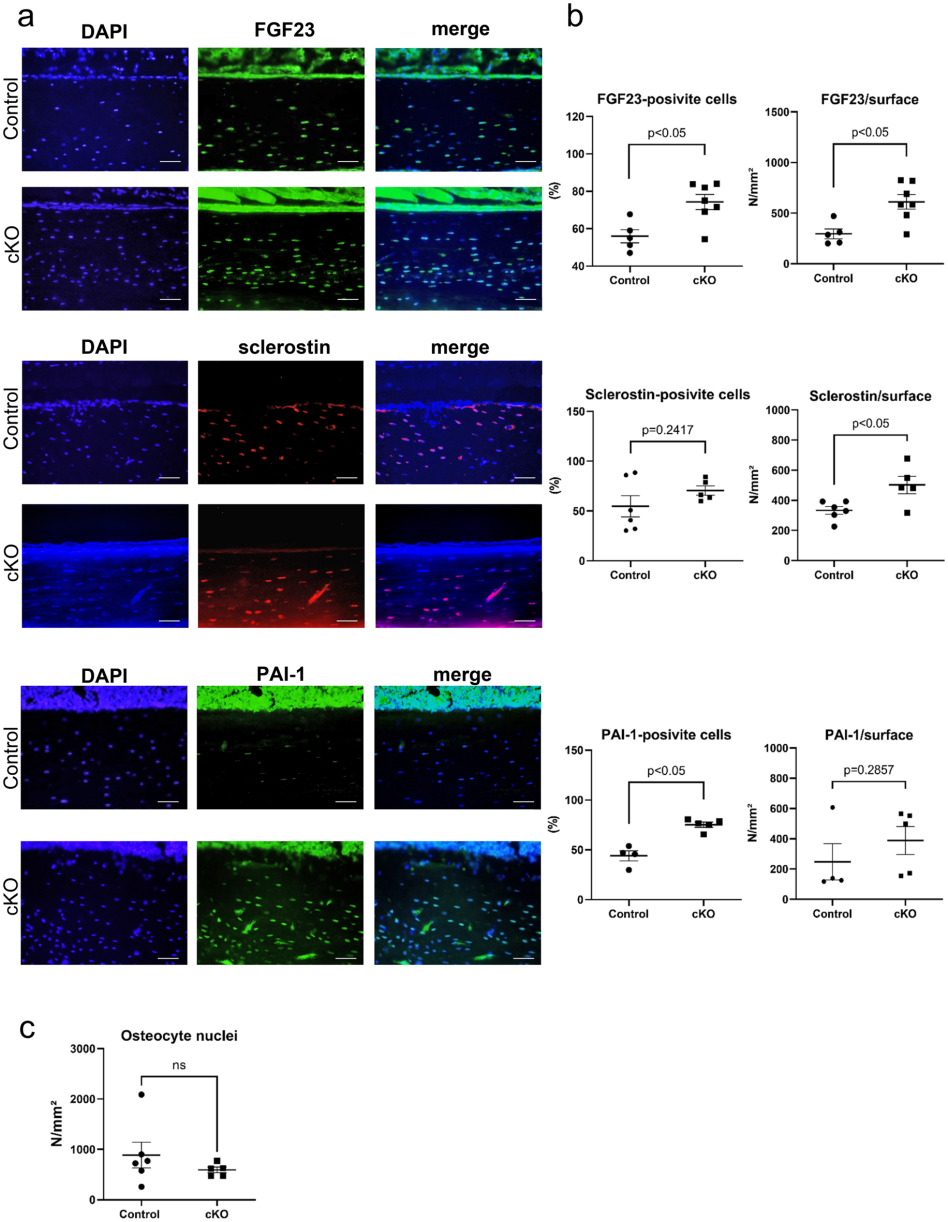
Sclerostin, FGF23, and PAI-1 protein expression is reduced in the cortical bone of cKO mice. (**a**) Representative images of FGF23, Sclerostin, and PAI-1 immunostaining in the cortical bone of the control and cKO mice (scale bar, 100 μm). (**b**) Quantification of immuno-positive cells. Left panels show immuno-positive osteocyte number/total osteocyte number. Right panels show immuno-positive osteocyte number/total cortex area. (**c**) Quantification of osteocyte nuclei in the cortical bone of control mice and cKO mice. Data represent mean ± SD for eight mice/group. *P < 0.05. cKO: DMP1cre::*Sirt6f.*/f mice.

Immunohistochemistry of the cortical bone was performed to detect the presence of SOST, FGF23, and PAI-1. The percentage of SOST-, FGF23-, and PAI-1-positive cells relative to total osteocytes was higher in cKO mice than in the control mice (Fig. 3a,b). These results indicate that SIRT6 deficiency stimulates the expression of SOST, FGF23, and PAI-1 in osteocytes. Osteocyte number was quantified by HE-stained cortical bone samples and showed no significant difference between wild type and cKO (Fig. 3c).

SIRT6 suppresses cellular senescence through multiple factors^20–24^, and PAI-1 is not only a marker of cellular senescence but is also sufficient for the induction of senescence^25^. Thus, cKO mice were crossed with PAI-1-null mice (*DMP-1*cre::*Sirt6f*^*/f*^::*Pai-1*−/− mice, cPKO mice) to investigate the role of PAI-1 in the phenotype of cKO mice. The cPKO mice grew normally, and their body weights were comparable with those of cKO and control mice (Supplementary Fig. 1).

### qPCR analysis of cortical bone in cPKO mice

**Figure 4:**
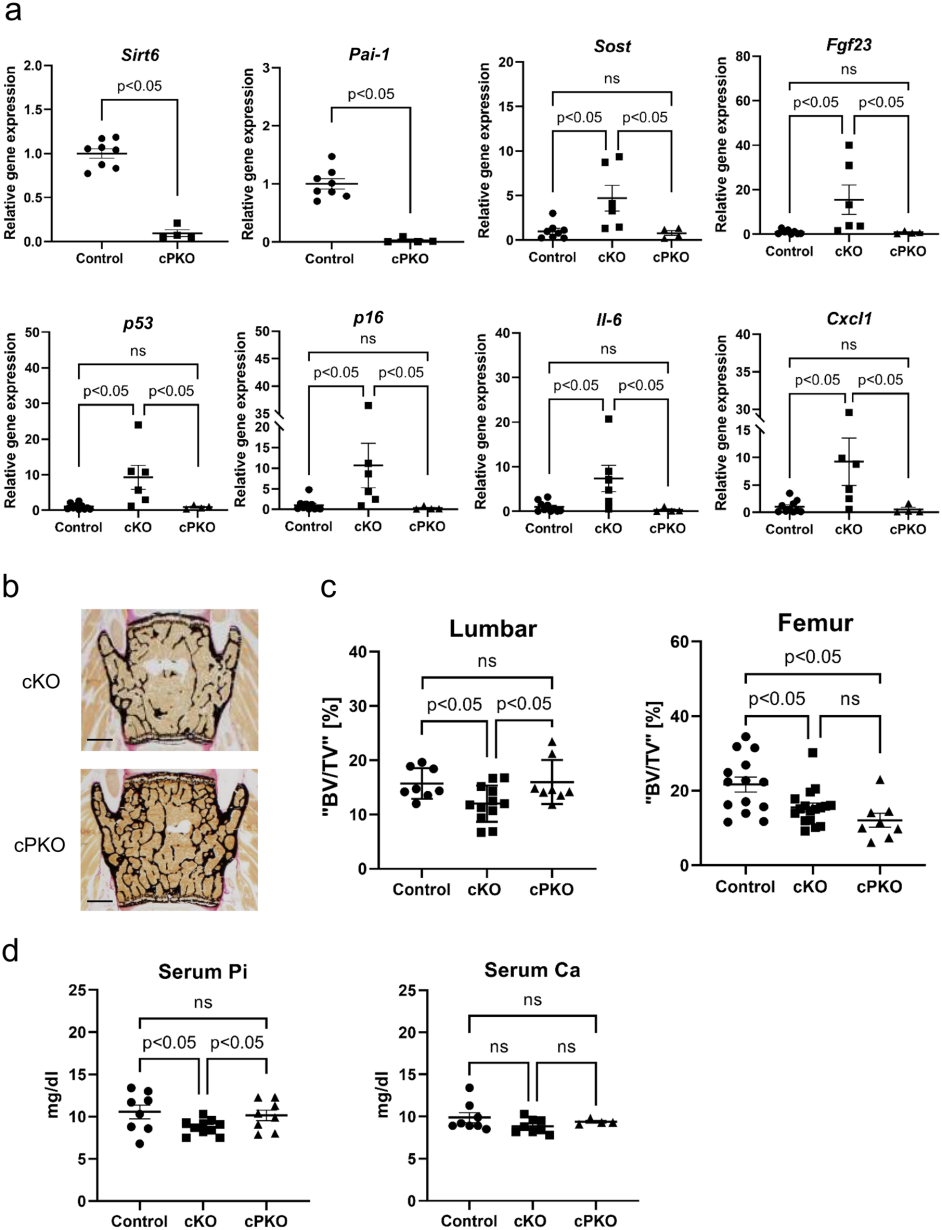
PAI-1 deficiency ameliorates SIRT6 deficiency-induced phenotypes. (**a**) qPCR analysis of the indicated genes in the cortical bone of cKO, cPKO, and the control mice. (**b**) Representative images of the lumbar spine from cKO, and cPKO mice (scale bar, 1 mm). (**c**) Values for BV/TV in the lumber bones (evaluated by histological analysis) and femoral bones (evaluated by micro-CT analysis). (**d**) Serum levels of phosphate and calcium in the control, cKO, and cPKO mice. Data represent mean ± SD. *P < 0.05. *cKO* DMP1cre::*Sirt6f*/f mice, *cPKO* DMP1cre::*Sirt6f*/f::PAI-1−/− mice; *BV* bone volume; *TV* trabecular bone volume.

qPCR analysis indicated that the mRNA expression of *Sirt6* and *Pai-1* were knocked out in the cortical bone samples of cPKO mice (Fig. 4a). We used qPCR analysis to investigate the expression of *Sost* and *Fgf23* in the cortical bones from cKO and cPKO mice and found that *Sost* and *Fgf23* mRNA levels were significantly reduced in cPKO compared to cKO mice (Fig. 4a). Thus, PAI-1 deletion clearly reduced the upregulation of these genes cause by SIRT6 deficiency. Similarly, the expression of *p53*, *p16, Il-6* and *Cxcl1*, was decreased in cPKO mice compared to cKO mice (Fig. 4a).

### Evaluation of bone parameters and serum phosphate levels in cPKO mice

Histological analyses showed higher bone volume in cPKO mice than in cKO mice in the lumber bone (Fig. 4b,c). In contrast, micro-CT analyses of distal femur showed the difference of BV/TV between cKO miceand cPKO mice was not significant. Reduced phosphate levels in cKO mice were mitigated by the deletion of PAI-1 in cPKO mice (Fig. 4d), whereas serum Ca^2+^ levels were comparable between cKO mice and cPKO mice (Fig. 4d).

### qPCR analysis of Sirt6 knockdown in MLO-Y4 cells

**Figure 5:**
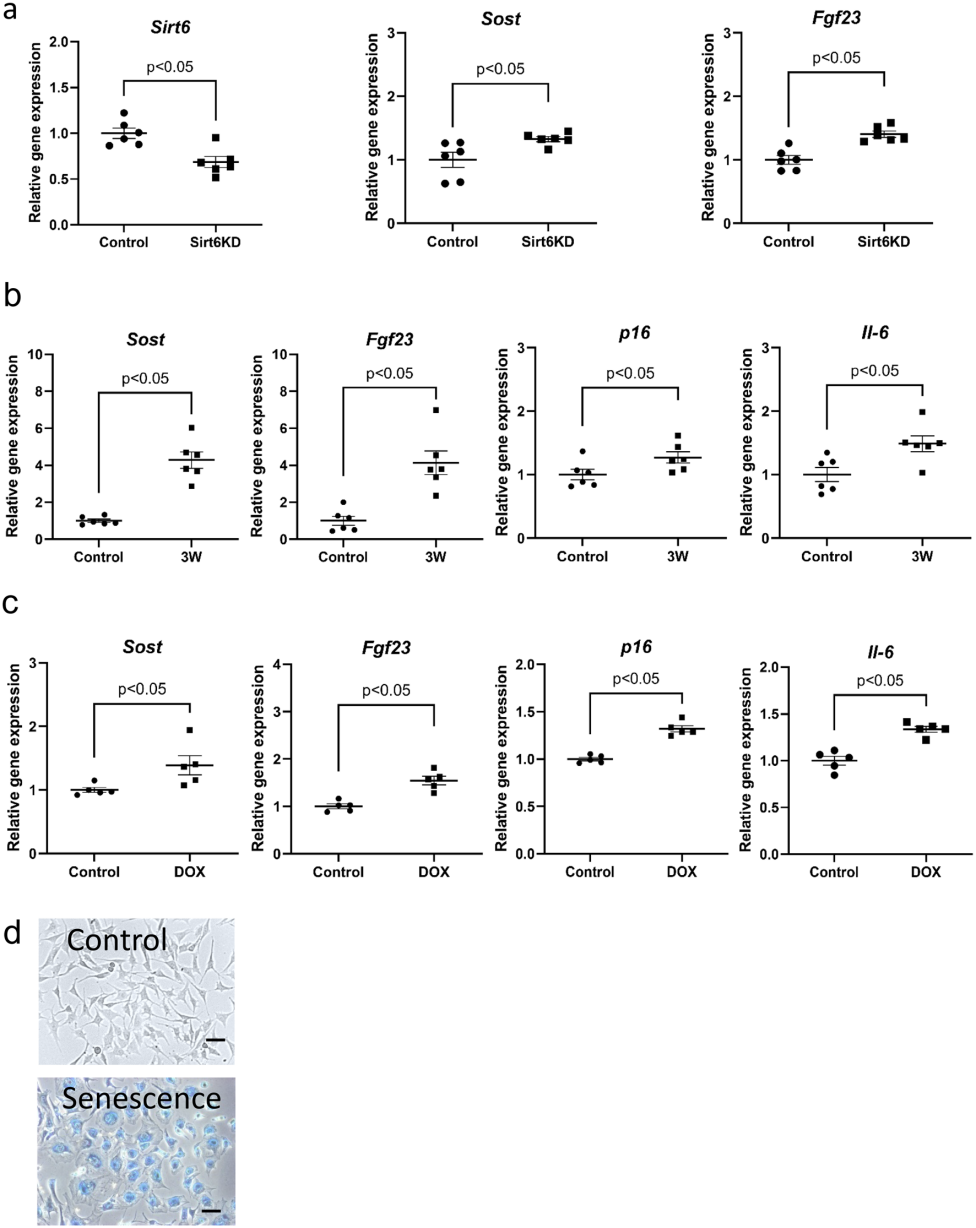
*Fgf23* and *Sost* mRNA expression is increased in SIRT6KD or senescent MLO-Y4 cells. (**a**) qPCR analysis of the indicated genes in SIRT6KD and control MLO-Y4 cells. (**b**) qPCR analysis of the indicated genes in MLO-Y4 cells cultured for three weeks after confluent. (**c**) qPCR analysis of the indicated genes in MLO-Y4 cells cultured with or without doxorubicin. (**d**) Representative images of SA-β gal in senescence-induced MLO-Y4 cells and control cells (scale bars, 20 µm). Data represent mean ± SD for five samples/group. *P < 0.05. *DOX* doxorubicin, *SIRT6KD* *Sirt6* knockdown using siRNA.

To investigate whether SIRT6 directly regulates *Sost* and *Fgf23* in osteocytes, Sirt6 was knocked down by siRNA in osteocyte-like MLO-Y4 cells. qPCR analysis indicated that *Sost, Fgf23* and *Pai-1* expression increased due to SIRT6 deficiency (Fig. 5a). This suggests that SIRT6 regulates *Sost, Fgf23* and *Pai-1* expression.

### qPCR analysis of senescent MLO-Y4 cells

To address the role of cell senescence in the expression of *Sost* and *Fgf23*, senescence was induced in MLO-Y4 cells using two different methods: long-term (21 days) culturing after confluency (Long-Term Confluent; LTC)^26,27^, and culturing under doxorubicin administration^28^. qPCR analysis revealed that *Sost* and *Fgf23* mRNA expression was stimulated in MLO-Y4 cells by both LTC and doxorubicin treatments, and the increase in *Sost* and *Fgf23* mRNA levels occurred concurrently with the upregulation of senescence marker gene and SASP marker gene, such as *p16* and *Il-6* (Fig. 5b,c). MLO-Y4 cells exhibited an increase in senescence-associated β-galactosidase (SAβ-gal) expression after induction (Fig. 5d).

### HIF-1α activation enhances Fgf23 expression

**Figure 6:**
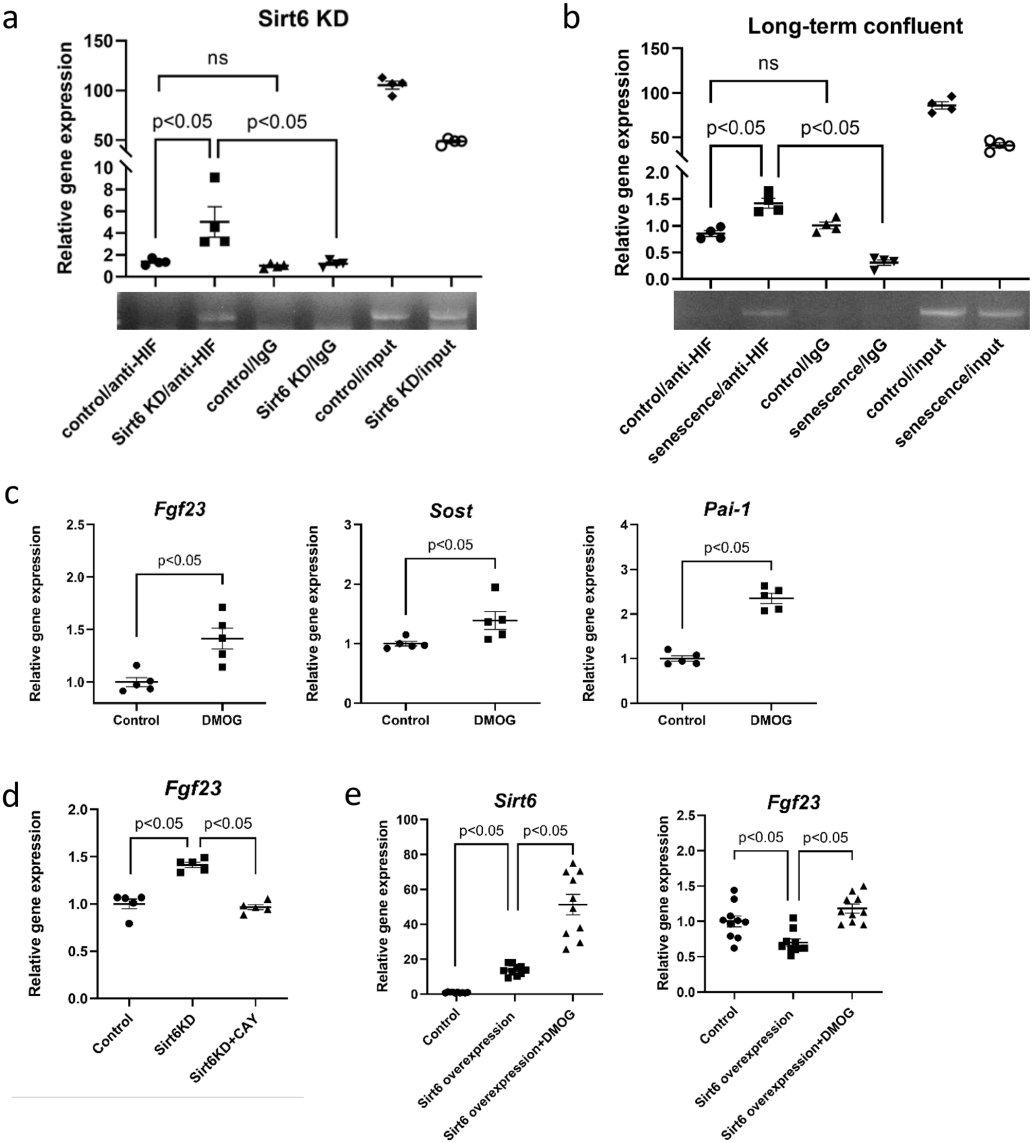
HIF-1α bound enhancer region of *Fgf23* upon SIRT6KD or senescence induction in MLO-Y4 cells. (**a**) ChIP assay analysis of SIRT6KD MLO-Y4 cells using the CRISPR/Cas9 system and (**b**) senescence-induced MLO-Y4 cells using long-term confluency, and respective control cells. The displayed data are representative of three experiments. (**c**) qPCR analysis of *Fgf23, Sost* and *Pai-1* in MLO-Y4 cells cultured with or without DMOG (HIF-1α activator). (**d**) qPCR analysis of *Fgf23* in SIRT6KD MLO-Y4 cells cultured with or without CAY10585 (HIF-1α inhibitor). (**e**) qPCR analysis of *Sirt6* and *Fgf23* in SIRT6 overexpressed MLO-Y4 cultured with or without DMOG. Data represent mean ± SD for five samples/group. *P < 0.05. *DMOG* N-(2-methoxy-2-oxoacetyl) glycine methyl ester; *SIRT6KD* *Sirt6* knockdown using the CRISPR/Cas9 system.

We investigated the mechanisms underlying the regulation of *Fgf23* transcription. There is a novel enhancer of *Fgf23* at -16 kb upstream of the transcriptional start site^29^. Additionally, the transcription factor HIF-1α activates *Fgf23* transcription in osteoblast-like cell lines^30^. SIRT6 directly binds to HIF-1α, preventing its activity^31^. Thus, we focused on the role of SIRT6 and cell senescence in HIF-1α-mediated *Fgf23* transcription. We examined the effect of SIRT6 deletion on the binding of HIF-1α to the *Fgf23* enhancer with a chromatin immunoprecipitation (ChIP) assay. The HRE (Hypoxia Response Element) identified in silico from the enhancer region located 16 kb upstream of the *Fgf23* transcription start site was used as a bait (Fig. 6a,b). HIF-1α protein was detected at low levels with the *Fgf23* enhancer DNA under basal conditions (Fig. 6a). However, when *Sirt6* was knocked out using the CRISPR/Cas9 system in MLO-Y4 cells, HIF-1α binding to the *Fgf23* enhancer sequence increased significantly (Fig. 6a). This finding suggests that SIRT6 regulates the binding of HIF-1α to the enhancer region of *Fgf23* in a cell-senescence-independent manner. We then investigated the binding of HIF-1α to the *Fgf23* enhancer in senescent cells. When senescence was induced in MLO-Y4 cells after 21 days of culture without passage, HIF-1α binding to the *Fgf23* enhancer increased significantly compared with that in MLO-Y4 cells under basal conditions (Fig. 6b). These data indicate that HIF-1α binds to the *Fgf23* enhancer region of MLO-Y4 cells in a senescence-dependent manner as well.

To evaluate the role of HIF-1α in the regulation of *Fgf23* expression, MLO-Y4 cells were cultured with an HIF-1α activator or inhibitor. The expression of *Fgf23*, along with that of *Pai-1*, was significantly increased in MLO-Y4 cells following the administration of N-(2-methoxy-2-oxoacetyl) glycine methyl ester (DMOG), a prolyl 4-hydroxylase (P4H) inhibitor, which increases HIF-1α levels by inhibiting HIF-1α prolyl hydroxylase (HIF-PH) (Fig. 6c). The expression of *Sost* was also increased by DMOG administration (Fig. 6c). When MLO-Y4 cells were cultured with CAY10585, an HIF-1α inhibitor that suppresses the transcription of HIF-1α target genes, *Fgf23* expression was not enhanced even when *Sirt6* was knocked down (Fig. 6d). In contrast, when SIRT6 was overexpressed in MLO-Y4 cells, the expression of *Fgf23* and *Sost* was suppressed, and these effects were cancelled by DMOG (Fig. 6e). These data indicate that HIF-1α activation is indispensable for *Fgf23* upregulation due to SIRT6 deficiency or senescence.

### Evaluation of bone parameters in aged PAI-1−/− mice

**Figure 7:**
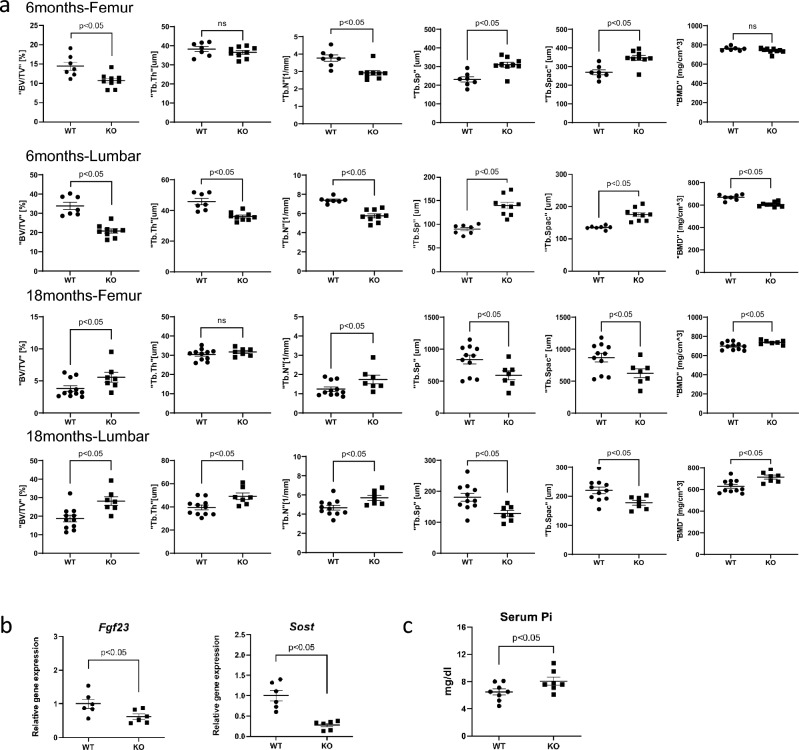
PAI-1-deficient mice have ameliorated age-related bone loss. (**a**) Micro-CT analysis of lumbar bone or femoral bone of wild-type (WT) or PAI-1KO (KO) mice in the indicated ages. (**b**) qPCR analysis of the indicated genes in 18-month-old WT, and 18-month-old PAI-1KO mice. (**c**) Serum phosphate levels in 18-month-old WT and 18-month-old PAI-1 KO mice. Data represent mean ± SD. *P < 0.05. *DOX* doxorubicin, *DMOG* N-(2-methoxy-2-oxoacetyl) glycine methyl ester, HIF-1 activator; *SIRT6KO* Sirt6 knockout, *CAY* CAY10585, a HIF-1 inhibitor; *BV/TV* bone volume/tissue volume; *Tb.th* trabecular bone thickness; *Tb.sp* trabecular bone separation; *Tb.Spac* trabecular bone spacing; *Tb.N* trabecular bone number, *BMD* bone mineral density.

To verify whether PAI-1 deficiency prevents age-related changes in bone tissues and mRNA transcripts, we compared bone tissues from aged PAI-1-deficient (PAI-1KO) and wild-type mice. At 6 months of age, the BV/TV of PAI-1KO was lower than that of WT and the trabecular number, trabecular separation, and trabecular spacing were higher in both the femur and lumbar spine. BMD was comparable in the lumbar spine, but PAI-1KO was lower in the femur. Conversely, at 18 months after birth, PAI-1KO was higher for BV / TV and trabecule number in both femur and lumbar spine, and PAI-1KO was lower for trabecular separation and trabecular spacing. BMD was also higher in PAI-1KO (Fig. 7a). Analysis of the cortical bone tissue by qPCR indicated that the mRNA expression of *Fgf23* and *Sost* decreased in PAI-1-KO mice compared with those in littermates at 18 months after birth (Fig. 7b). Serum phosphate levels were elevated in 18 months-old PAI-1-KO mice compared with those in littermates (Fig. 7c).

### Evaluation of the expression of FGF23, SOST and PAI-1 mRNA in human bone

**Figure 8:**
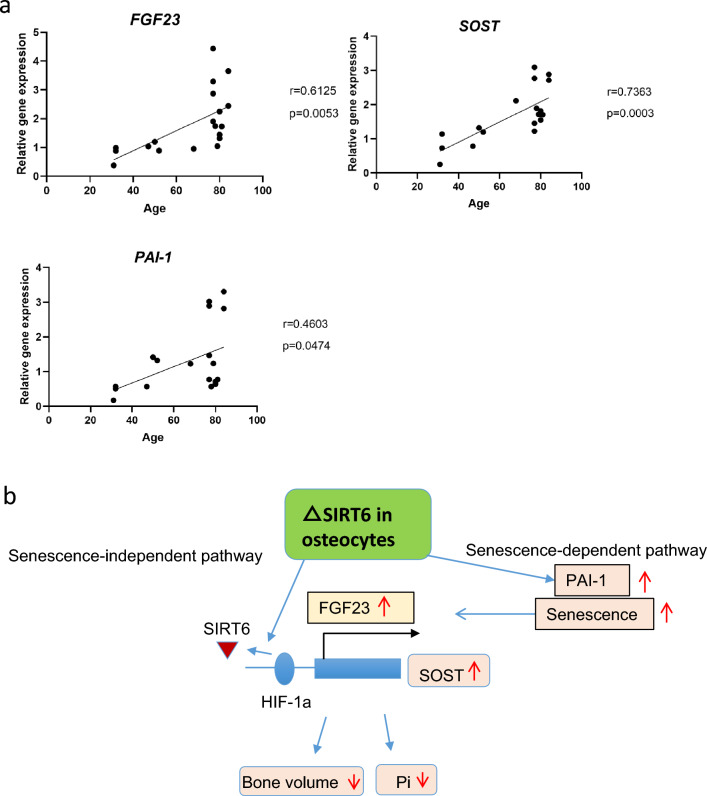
Evaluation of FGF23, SOST and PAI-1 in human bone. (**a**) qPCR analysis of the indicated genes in human bone. mRNA expression of *FGF23*, *SOST* and *PAI-1* were positively correlated with age. (**b**) Proposed SIRT6 signaling pathwaysWe propose two signaling pathways: senescence-dependent and -independent. SIRT6 directly binds HIF-1α and inhibits transcription of its target gene. SIRT6 inactivation results in HIF-1α binding to the *Fgf23* consensus motif within its enhancer sequence in MLO-Y4 cells. SIRT6 inactivation enhances the expression of PAI-1, which induces cell senescence. Induction of senescence in MLO-Y4 cells also increases the binding of HIF-1α to the *Fgf23* enhancer sequence. *Sost* expression also increases as a result of SIRT6 deletion through the senescence-dependent and -independent pathways.

Finally, to analyze the relationship between the expression of FGF23, SOST, and PAI-1 in human bone and age, q-PCR analysis was performed using bone samples from the femoral neck bone taken during hip arthroplasty. The results showed that the expression levels of *FGF23*, *SOST*, and *PAI-1* were significantly correlated with age of donors (Fig. 8a).

## Discussion

The regulatory mechanisms of bone mass in aged animals are poorly understood. We aimed to investigate the role of SIRT6, a longevity-associated factor, in osteocytes. We found that Sost, Fgf23, expression was increased in SIRT6-deficient osteocytes, leading to a decrease in osteoblasts and an increase in osteoclasts, resulting in decreased bone mass. In addition, PAI-1, whose expression was promoted by SIRT6 deficiency, was actively involved in bone metabolism, indicating that PAI-1 deficiency suppresses age-related bone loss. Thus, we showed that induction of senescence by SIRT6 deletion was one of the causes of age-related changes in bone metabolism. Based on the results of this study, we proposed two signaling pathways downstream of SIRT6, a senescence-dependent and a senescence-independent pathway involved in the regulation of SOST and FGF23 (Fig. 8b). The relationship between degenerative cellular changes associated with aging and bone loss has been studied. Aging decreases cellular mitochondrial activity and increases oxidative stress. Genetically engineered mice with mitochondrial dysfunction or loss of mitochondrial homeostasis exhibit osteoporosis^32,33^. Mice lacking osteocyte-specific mitochondrial superoxide dismutase 2 (SOD2) exhibit osteocytopenia and osteoporosis with increased ROS levels^34^.

Among the studies on the association between sirtuin genes and bone metabolism, SIRT1 has been the most studied. SIRT1 promotes mitochondrial production through deacetylation of PGC-1a and inactivation of HIF-1a, and decreased SIRT1 activity leads to mitochondrial depletion^35^. Mice lacking SIRT1 specific in osteoblasts or osteoclasts both exhibit bone loss via increased NF-kB activity^36^. In mice in which SIRT1 is stabilized in osteocytes, *Sost* transcription is inhibited^37^. Combined with the results of this study, it is clear that both SIRT1 and SIRT6 regulate SOST expression. Rats lacking SIRT6 specifically in bone marrow mesenchymal stem cells exhibited suppressed osteoblast differentiation, whereas rats overexpressing SIRT6 exhibited the opposite phenotype. Both suggested that NF-kB regulation may be at least partially involved in bone metabolism^38^. Although SIRT6 global knockout mice exhibit low-turnover osteoporosis^5^, osteoblast-specific SIRT6-deficient mice driven by the osteocalcin promoter show reduced bone mass due to increased osteoclasts associated with reduced OPG expression^39^. Interestingly, bone formation parameters were not affected in these mice. Mice lacking SIRT6 in hematopoietic cells, including osteoclast precursors, had reduced osteoclast numbers and increased bone mass; SIRT6 formed a complex with B lymphocyte-induced maturation protein-1 (Blimp1) and suppressed the expression of osteoclast differentiation inhibitors such as Mafb^40^.

SIRT6 deletion induces cell senescence^6,24^ SIRT6 binds to telomeres and prevents cell senescence by preserving telomere function through H3K9 deacetylation^24,41^. Furthermore, SIRT6 deficiency enhances *Pai-1* expression, which induces replicative senescence downstream of p53^3,4,21,25^. cKO mice showed increased *Fgf23* expression and osteocyte senescence, and *Pai-1* depletion in cKO mice led to the normalization of *Fgf23* expression. Similarly, two different methods for the induction of cell senescence (LTC and doxorubicin treatment) stimulated *Fgf23* expression. In cells undergoing senescence, activation of the p53-p21 and p16-RB pathways are induced. p16 and p21 have different target CDKs, and each of them alone has a weak ability to activate RB protein. However, the simultaneous action of p16 and p53-p21 cause the RB protein to be permanently activated and arrest the cell cycle progression^42^. In this study, we showed that both p16 and p53 expression were upregulated in SIRT6-deficient osteocytes, and that PAI-1 deletion suppressed these expressions. These data suggested that SIRT6 regulated cell senescence induction mechanisms at least in part through the regulation of PAI-1 expression. Senescent cells express inflammatory cytokines, such as IL-6, PAI-1, and CXCL1, as part of the senescence-associated secretory phenotype (SASP) response^41^. IL-6 stimulates *FGF23* promoter activity through STAT3 in osteoblast-like UMR106 cells^43^*.* NF-κB signaling plays a critical role in the induction of SASP^44^. NF-κB activation results in *FGF23* upregulation downstream of PKC or p38 MAPK^45,46^. These data indicate that senescence plays a role in *FGF23* regulation. In hepatic cells, SIRT6 directly binds to HIF-1α and inhibits the transcription of its target gene, *Glut-1*^31^. Through the deletion of *Sirt6* in MLO-Y4 cells, we showed that HIF-1α was able to bind to the *Fgf23* enhancer via a consensus motif within its sequence, which promotes *Fgf23* transcription (Fig. 6a).

Senescent cells have a causal role in aging-related osteoporosis, and the depletion of senescent cells in the bone ameliorates aging-related osteoporosis^18^. Senescent cell-depleted mice present a bone tissue phenotype characterized by a reduced number of osteoclasts and an increased number of osteoblasts, both of which have been linked to the suppression of *Sost* expression^18^. These data, together with the findings of the present study, indicate that cell senescence, regulation of *Sost* expression, and possibly SASP, play important roles in aging-related osteoporosis. Epigenetic regulation of *Sost* controls sclerostin levels, which correlates with bone density and fracture rates^47^. The promoter region of *Sost* has a CpG-rich domain, which contributes to the transcriptional regulation of *Sost*^48^. A study of postmenopausal women showed lower *Sost* mRNA levels in patients with osteoporosis and significantly increased CpG methylation in the *Sost* promotor region^48^. SIRT6 has histone deacetylase (HDAC) activity, and so it may regulate the expression of *Sost* through deacetylation of the promoter region; however, the role of the HDAC activity of SIRT6 was not elucidated in this study.

PAI-1 is a novel regulator of FGF23 metabolism^49^. HIF-1 promotes PAI-1 transcription, thereby increasing its expression. In osteocytes, hypoxia or age-related decrease in SIRT6 activity may promote *Pai-1* expression through HIF-1 activation, leading to osteocyte senescence. In fact, there was an increase in *Pai-1* expression in cKO mice.

The candidate senescence-independent factors for the regulation of *Sost* downstream of SIRT6 are HIF-1 and NF-κB*. Sost* expression is induced by hypoxia through HIF-1α activation in MC3T3-E1 osteoblastic cells^50^. HIF-1α activates *Sost* transcription by binding to its promoter in MC3T3-E1 cells^50^. Additionally, SOST levels are elevated by hypoxia in human Saos-2 osteogenic sarcoma cells^51^. Consistently, we reported that *Sost* expression was induced by SIRT6 deletion or cell senescence in the MLO-Y4 osteocyte-like cell line in a HIF-1α-dependent manner. Together, these results suggest that SIRT6 may bind to HIF-1α and regulate *Sost* expression via its HDAC activity. TNFα stimulates *Sost* expression through an NF-κB-dependent mechanism in MLO-Y4 cells. NF-κB directly binds to NF-κB binding elements on the *Sost* promoter to induce *Sost* transcription^52^. SIRT6 is also a negative regulator of NF-κB signaling^23^. Thus, NF-κB activation may be involved in *Sost* upregulation in cKO mice; however, the role of NF-κB was not elucidated in this study.

In conclusion, we showed that osteocyte SIRT6 regulates bone volume and phosphate metabolism by modulating *Sost* and *Fgf23* expression. This novel pathway explains the association between *Fgf23* and *Sost* and aging-related disorders. Furthermore, PAI-1 deficiency, along with the normalization of *Sost* expression, in cKO mice abolished the effects of SIRT6 deletion in bone tissues. A study of the Berne Amish community members, including those that are carriers of the null *SERPINE1* gene that encodes PAI-1, revealed that a rare loss-of-function mutation in *SERPINE1* prolongs human life and prevents age-related metabolic abnormalities^53^. Moreover, a small molecule PAI-1 inhibitor has been developed, and clinical trials are underway in humans^54^. We previously revealed that PAI-1 inhibition via a small molecule PAI-1 inhibitor prevented ovariectomy-induced bone loss in mice^55^. Nicotinamide mononucleotide (NMN) activates sirtuins, including SIRT1 and SIRT6, and its long-term administration corrects age-related metabolic disorders^56^. NMN administration promotes bone formation by preventing bone marrow mesenchymal stem cells from differentiating into adipocytes in aged mice^57^. NMN is confirmed to be safe in humans, and clinical studies regarding their use are being conducted^58^. Overall, these results support the potential therapeutic application of a SIRT6 agonist, such as NMN or a PAI-1 inhibitor, against aging-related disruptions of bone metabolism.

## Methods

All methods were carried out in accordance with ARRIVE guidelines.

### Reagents

The following reagents were purchased from the indicated sources: doxorubicin (Fujifilm, Tokyo, Japan), N-(2-methoxy-2-oxoacetyl) glycine methyl ester (R&D Systems, Minneapolis, MN, USA), and CAY10585 (Abcam Biochemicals, Cambridge, UK).

### Animals

All animal experiments were approved by the Animal Care and Use Committee of Tokyo Medical and Dental University and were carried out in accordance with the approved guidelines (approval number: A2018316). All mice were allowed unrestricted activity and given access to standard rodent food pellets (Labo MR Stock, Nosan, Tokyo, Japan) and tap water ad libitum. *Sirt6f*^/+^ mice (FVB.129S6(Cg)-*Sirt6*tm1.1Cxd/J) were obtained from the Jackson Laboratory (Bar Harbor, ME, USA). *Dmp1*-Cre (B6N.FVB-Tg (*Dmp-1*-Cre)1Jqfe/BwdJ) mice were kindly supplied by Dr. Shu Takeda from the Endocrine Center, Toranomon Hospital. *Sirt6f*^*/f*^ mice were backcrossed for at least ten generations with C57/BL6J mice (CLEA Japan Inc., Tokyo, Japan) and then crossed with *Dmp-1*-Cre mice to generate *Dmp-1*-Cre::*Sirt6f*^/+^ mice, and their progeny were intercrossed to obtain cKO mice. PAI-1 ± mice (C57BL6/J background) were obtained from the Jackson Laboratory. PAI-1 ± mice were crossed with *Dmp-1*-Cre::*Sirt6f*^/+^ mice to obtain *Dmp-1*-Cre *Sirt6f*^/+^::*Pai-1* ± mice. cPKO mice were obtained by crossing *Dmp-1*-Cre::*Sirt6f*^/+^::PAI-1 ± mice. Male mice were euthanized at 20 weeks of age using an overdose of isoflurane inhalation followed by cervical dislocation. Male PAI-1−/− mice and their littermates were grown for 72 weeks, and their samples were analyzed by micro-CT and qPCR analysis following euthanasia. None of the mice died during the experimental period.

### Histological and histomorphometric analysis

Calcein (Sigma-Aldrich, St. Louis, MO, USA) was injected subcutaneously for bone labeling five and two days prior to euthanasia. Blood and bone samples were collected at the time of euthanasia. The undecalcified samples of the spinal bone were sectioned, and the third and fourth lumbar vertebrae were stained using von Kossa and tartrate-resistant acid phosphatase (TRAP) staining, as previously described^59^. The OsteoMeasure Analysis System (OsteoMetrics, Decatur, GA, USA) was used for static and dynamic histomorphometric analyses following the nomenclature defined by the American Society for Bone and Mineral Research, as previously described^60^.

### Immunohistological analysis

Cortical bone sections were fixed in 4% paraformaldehyde on ice for 10 min. After washing with 1% T-PBS buffer for three times (5 min each), the sections were blocked with normal serum at room temperature for 1.5 h to reduce non-specific binding. Primary antibodies were applied and incubated at 4 °C overnight. The primary antibodies used were anti-FGF23 (R&D systems; catalog Number: MAB26291) (1:200), anti-sclerostin (R&D systems; catalog Number: AF1589) (1:200), and anti-PAI-1 (Abcam Biochemicals; catalog number: ab66705) (1:200). After washing with T-PBS buffer for three times (5 min each), the sections were incubated with fluorescent-labeled secondary antibodies at room temperature for 1.5 h. The secondary antibodies used were Goat Anti-Rabbit IgG (H + L), Mouse/Human ads-FITC (Southern Biotech, Birmingham, AL, USA; Catalog Number: AB_2795952) (1:200), donkey anti-goat IgG (H + L) Cross-Adsorbed Secondary Antibody, Alexa Fluor 594 (Thermo Fisher Scientific, Waltham, MA, USA, Catalog Number: A-11058) (1:200), and anti-Rat IgG2a Antibody Conjugate FITC (Bethyl Laboratories Inc., Montgomery, AL, USA, Catalog Number: A110-109F) (1:200). After washing with T-PBS buffer for three times (5 min each), the excess liquid was removed by gentle drying or wiping with a tissue, and the sections were mounted with a coverslip and sealed with DAPI. The fluorescent images were captured and analyzed using Fiji software (developed by Wayne Rasband, National Institutes of Health, Bethesda, MD, USA). Quantification of immune-positive cells was performed by three independent raters. There was no predominant inter-rater variability in the results of the three evaluations.

### Micro-CT analysis

Two-dimensional images of the distal femur and lumbar spine were obtained through micro-CT analysis (Comscan, Yokohama, Japan). The following three-dimensional morphometric parameters were determined using TRI/3D-BON software (RATOC, Tokyo, Japan): bone morphometric analysis of femoral bones performed at a region of 0.2 to 1 mm above the distal growth plates of the femora, bone volume/tissue volume (BV/TV), trabecular bone thickness (Tb.Th), Tb.N, Tb.Sp, and Tb.Spac for trabecular bone analyses^61^. The mineralized tissue volumes were measured using a calibration curve obtained from the BMD phantom.

### Cell culture and gene knockout with the CRISPR/Cas9 system and small interfering RNA (siRNA)

The mouse osteocyte-like MLO-Y4 cell line was purchased from RIKEN Cell Bank (Tsukuba, Japan). MLO-Y4 cells were cultured in alpha minimal essential medium (αMEM) containing 2.5% fetal bovine serum (FBS) and 2.5% calf serum (CS; Gibco Bovine Serum, Thermofisher scientific, Massachusetts, USA) with medium change every 3 days as described previously^62^. MLO-Y4 cells were transferred to 6-well plates and, at 70% confluency, were transfected with 50 nM *Sirt6* siRNA or scrambled siRNA with HiPerFect (Qiagen, Valencia, CA, USA) in medium supplemented with 10% FBS for 24 h. The target sequence of *Sirt6* siRNA was 5′-GAAGCUCCCAAUGCAAUAAAU-3′ (forward) and 5′-UUAUUGCAUUGGGAGCUUCUG-3′ (reverse).

The CRISPR/Cas9 knockout (catalog number: sc-424467-KO-2), HDR (catalog number: sc-424467-HDR-2), and control (catalog number: sc-418922) were purchased from Santa Cruz Biotechnology (Dallas, TX, USA). *Sirt6* knockout was performed according to the manufacturer’s instructions.

### Senescence induction

For senescence induction by long-term confluency, MLO-Y4 cells were grown at confluency for 21 days without subculture^63,64^. αMEM medium supplemented with 2.5% FBS, 2.5% CS (Gibco Bovine Serum), and 1% antibiotics (Gibco Antibiotic–Antimycotic (100X); catalog number: 15240062) was used to grow MLO-Y4 cells. For DNA damage-induced cellular senescence, MLO-Y4 cells were treated with 50 nM doxorubicin, and 48 h later, mRNA was harvested for qPCR^65^. SA-β-gal activity was detected using the Senescence Detection Kit according to the manufacturer’s instructions (BioVision, CA, USA, catalog number: K320-250).

### Sirt6 overexpression assay

MLO-Y4 cells were cultured in α-minimum essential medium (αMEM) supplemented with 2.5% fetal bovine serum (FBS) and 2.5% bovine calf serum (CS; Gibco Bovine Serum, Thermofisher Scientific, Massachusetts, USA). Cells were seeded onto a 6-well plate and transfected at 80% confluency. Control group was transfected with pCMV-entry vector (1.5 μg/well) (Sirt6 (NM_181586) Mouse Tagged ORF Clone; CAT#: MG204964), while the overexpression and overexpression + DMOG groups were transfected with pCMV-sirt6 vector (1.5 μg/well) (pCMV6-Entry Mammalian Expression Vector; CAT#: PS100001). Lipofectamine2000 reagent was used according to the manufacturer's instructions to form Lipofectamine2000-DNA complexes. The mixture was incubated separately in Opti-MEM® and then the two solutions were mixed to form the complex. The Lipofectamine2000-DNA complex was added to the αMEM medium containing MLO-Y4 cells and incubated for 18 h. The medium was then replaced with fresh αMEM medium supplemented with 2.5% FBS, 2.5% CS, and 1% antibiotic–antimycotic (Gibco Antibiotic–Antimycotic (100X); Catalog #: 15240062). DMOG (1 mM) was added to the overexpression + DMOG group. After 24 h, mRNA was harvested for qPCR analysis.

### Chromatin immunoprecipitation assay

The EpiQuik ChIP kit (Epigentek Group Inc., NY, USA) was used for the ChIP assay, and it was performed according to the manufacturer’s instructions. To evaluate the binding of HIF-1α to the *Fgf23* enhancer^29^, PCR was carried out using a Thermal Cycler 9700 (Applied Biosystems, Foster City, CA, USA) according to a standard procedure. The primer sequences used were

- 5′-GTCAAGTGAGTCCGGCTTCA-3′ (forward) and
- 5′-CCGAGCCAGGACTTTCCTTT-3′ (reverse).

### Human samples

The Ethics Committee of Tokyo Medical and Dental University approved this study (approval number: M2000-2121). All methods were carried out in accordance with the guidelines and regulations of the Ethics Committee of Tokyo Medical and Dental University. All the donors provided written informed consent to participate in the study. Bone samples were harvested under informed consent from the femoral neck of patients (n = 19) with hip osteoarthritis or femoral hip fracture scheduled to undergo total hip arthroplasty or surgical fixation. Exclusion criteria included a history of steroid administration, inflammatory arthritis, autoimmune disease, metastatic cancer, osteonecrosis of femoral head, endocrine disorders, or bone-related disorders.

### Statistical analysis

Data are presented as mean ± standard deviation. Statistical analyses of the quantitative measures among the three groups were performed using one-way ANOVA. To assess the significance of differences between groups, a two-tailed Student’s *t*-test was employed. Statistical significance was set at P < 0.05. Pearson linear regression was used to determine the degree of association between mRNA expression of *FGF23*, *SOST*, *PAI-1* and age of donors. The linear regression coefficient R were reported. Values of P < 0.05 were accepted as significant.

## Supplementary Information

Supplementary Figures.

## Data Availability

The datasets used and/or analyzed during the current study are available from the corresponding author upon reasonable request.
