# Supplementary material for: SIRT6-PAI-1 axis is a promising therapeutic target in aging-related bone metabolic disruption
Source: Sci Rep. 2023 May 17;13:7991. doi: 10.1038/s41598-023-33297-7 (PMC10192395; doi:10.1038/s41598-023-33297-7)
Supplement: Supplementary file 1 — Supplementary Figures. [file 41598_2023_33297_MOESM1_ESM.docx]

**SIRT6-PAI-1 axis is a promising therapeutic target in aging-related bone metabolic disruption**

Alkebaier Aobulikasimu^1#^, Tao Liu^1#^, Jinying Piao^1^, Shingo Sato^1^, Hiroki Ochi^2^, Atsushi Okawa^1^, Kunikazu Tsuji^3^, Yoshinori Asou^3#^*

^1^Department of Orthopedics Surgery, Tokyo Medical and Dental University 1-5-45 Yushima Bunkyo-ku, Tokyo 113-8519, Japan

^2^Department of Rehabilitation for Movement Functions, Research Institute, National Rehabilitation Center for Persons with Disabilities, 4-1, Namiki, Tokorozawa-shi, Saitama 359-8555, Japan

^3^Department of Nano-Bioscience, Tokyo Medical and Dental University, 2-3-10, Kanda Surugadai, Chiyoda-ku, Tokyo 101-0062, Japan

^#^These authors contributed equally to this article.

**Author Email addresses and phone numbers**

Alkebaier Aobulikasimu, taiyang575@163.com; 81-3-3813-6111

Tao Liu, liuorth@tmd.ac.jp; 81-3-3813-6111

Jinying Piao, bokureh@tmd.ac.jp; 81-3-3813-6111

Shingo Sato, s.sato.0726@gmail.com; 81-3-3813-6111

Hiroki Ochi, hiroki.vet101@gmail.com; 81-3-3813-6111

Atsushi Okawa, okawa.orth@tmd.ac.jp; 81-3-3813-6111

Kunikazu Tsuji, tsuji.orj@tmd.ac.jp; 81-3-3813-6111

Yoshinori Asou, aso.orth@tmd.ac.jp; 81-3-3813-6111

*Corresponding author: Yoshinori Asou

Department of Nano-Bioscience, Tokyo Medical and Dental University

2-3-10, Kanda Surugadai, Chiyoda-ku, Tokyo 101-0062, Japan

Phone: 81-3-3813-6111

Email: aso.orth@tmd.ac.jp

**Supplementary data**


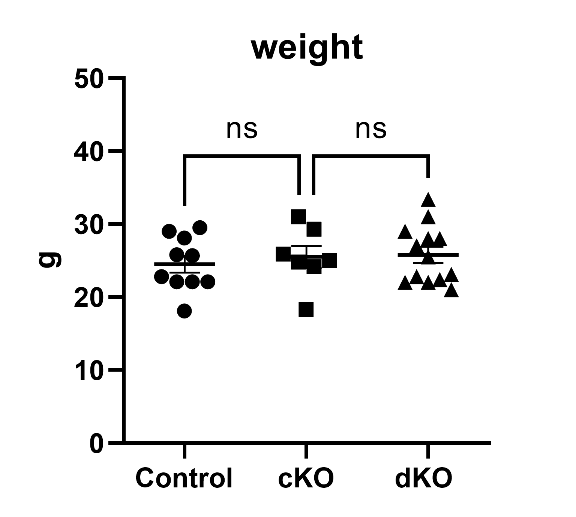


**Supplementary Figure 1**. Body weight of the control, cKO and cPKO mice at sacrifice.


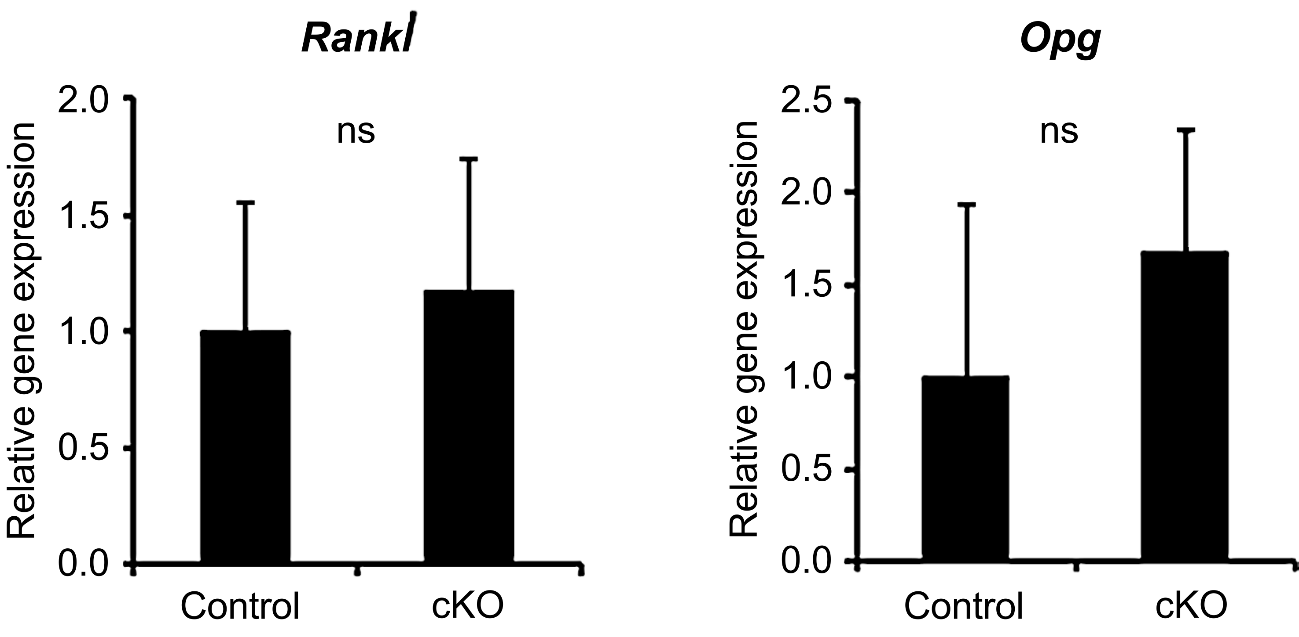


**Supplementary Figure 2**. qPCR analysis for *Rankl and Opg* in the control and cKO mice.
